# Chemotherapy with cytochalasin congeners in vitro and in vivo against murine models

**DOI:** 10.1007/s10637-014-0203-5

**Published:** 2015-01-07

**Authors:** Matthew Trendowski, Joan M. Mitchell, Christine M. Corsette, Christopher Acquafondata, Thomas P. Fondy

**Affiliations:** Department of Biology, Syracuse University, 107 College Place, Syracuse, NY 13244 USA

**Keywords:** Cytochalasin congeners, Lung carcinoma, Melanoma, Leukemia, Drug synergy

## Abstract

*Background* Despite inherent differences between the cytoskeletal networks of malignant and normal cells, and the clinical antineoplastic activity of microtubule-directed agents, there has yet to be a microfilament-directed agent approved for clinical use. One of the most studied microfilament-directed agents has been cytochalasin B, a mycogenic toxin known to disrupt the formation of actin polymers. Therefore, this study sought to expand on our previous work with the microfilament-directed agent, along with other less studied cytochalasin congeners. *Materials and Methods* We determined whether cytochalasin B exerted significant cytotoxic effects in vitro on adherent M109 lung carcinoma and B16BL6 and B16F10 murine melanomas, or on suspension P388/ADR murine leukemia cells. We also examined whether cytochalasin B, its reduced congener 21, 22-dihydrocytochalasin B (DiHCB), or cytochalasin D could synergize with doxorubicin (ADR) against ADR-resistant P388/ADR leukemia cells, and produce significant cytotoxicity in vitro. For in vivo characterization, cytochalasins B and D were administered intraperitoneally (i.p.) to Balb/c mice challenged with drug sensitive P388-S or multidrug resistant P388/ADR leukemias. *Results* Cytochalasin B demonstrated higher cytotoxicity against adherent lung carcinoma and melanoma cells than against suspension P388/ADR leukemia cells, as assessed by comparative effects on cell growth, and IC_50_ and IC_80_ values. Isobolographic analysis indicated that both cytochalasin B and DiHCB demonstrate considerable drug synergy with ADR against ADR-resistant P388/ADR leukemia, while cytochalasin D exhibits only additivity with ADR against the same cell line. In vivo, cytochalasins B and D substantially increased the life expectancy of mice challenged with P388/S and P388/ADR leukemias, and in some cases, produced long-term survival. *Conclusion* Taken together, it appears that cytochalasins have unique antineoplastic activity that could potentiate a novel class of chemotherapeutic agents.

## Introduction

Cytochalasins are secondary mold metabolites that have been shown in over three thousand publications to produce profound pleotypic effects on normal and neoplastic cells, and on tissues in culture. These effects appear to arise largely because of the ability of various cytochalasin congeners to alter microfilament structure and function, and therefore to affect the myriad of cell and tissue functions that are dependent on microfilament biochemistry.

Cytochalasins alter cell motility, adherence, secretion, drug efflux, deformability, morphology, and size, among many other effects. Their influence on cell adherence includes alteration of cell junctions leading to changes in tissue physiology and transport. Moreover, they inhibit plasma membrane division without similarly affecting nuclear division, thus producing multinucleated cells. For detailed references on cytochalasins, see comprehensive reviews [1, 2] and for specific references and further elaboration, see the preceding papers in this series [3, 4].

The multinucleation phenomenon is more evident in neoplastic cells and less so in their normal counterparts, potentially representing a neoplastic-targeting feature of cytochalasin mechanisms [5–12]. This apparent partial specificity of cytochalasin action on neoplastic cells is consistent with a separate body of evidence showing substantial differences in microfilament biochemistry between neoplastic and comparable non-neoplastic cells [13]. These differences in microfilament structures in turn may be related to key features of the neoplastic phenotype, including altered adherence, anchorage independent growth, invasiveness, and altered plasma membrane cytoskeletal interactions involving in part expression of oncogene products [14, 15].

It has been well documented that tumors often exhibit innate or acquired resistance to chemotherapeutic agents due to the overexpression of ATP-binding cassette (ABC) transporters, which carry a substantial variety of compounds across cellular compartments [14–16]. In particular, the plasma membrane-spanning proteins, permeability glycoprotein (P-gp) and multidrug resistance-associated protein (MRP), confer resistance to anthracyclines, taxanes, vinca alkaloids, and other bulky chemotherapeutic agents due to drug efflux catalyzed by the multi-drug resistance pump [17–19]. However, cytochalasin B efflux is not potentiated by overexpression of ABC transporters [20], a surprising finding given the structural bulk of the compound and its hydrophobicity. Due to its novel mechanisms of action, it may be possible to use cytochalasin B concomitantly with currently approved chemotherapeutic agents to reduce or even eliminate specific instances of drug resistance. This is particularly plausible for DNA-directed agents, because cytochalasin B potentiates multinucleation [2, 14]. Substantial multinucleation increases the likelihood of apoptosis, as it takes only a single nucleus to undergo programmed cell death before a chain reaction is triggered, culminating in the destruction of the cell [21]. P388/ADR murine leukemia is well known for its resistance to doxorubicin (trade name: Adriamycin, ADR) due to an overexpression of P-gp [22–24]. ADR is an anthracycline that intercalates DNA, thereby inhibiting topoisomerase II from relaxing DNA supercoiling during transcription [16].

As such, it is likely that cytochalasin B administered concomitantly with ADR will produce substantial drug synergy, potentially reversing ADR-resistance in P388/ADR cells. However, cytochalasin B also has the propensity to inhibit glucose transport [25–27]. Glucose transport inhibition has been shown to decrease drug efflux in vincristine-resistant murine erythroleukemia PC4 cell lines [28]. Using 21, 22-dihydrocytochalasin B (DiHCB), a congener that has very similar effects on microfilaments without notable glucose transport inhibition [29, 30], concurrently with ADR should help determine whether glucose transport inhibition has any bearing on cytochalasin/ADR drug synergy.

Although not as well-studied as cytochalasin B, its congener cytochalasin D has also demonstrated marked anticancer activity [31–33]. It has been observed in vitro that cytochalasin D is a much more potent inhibitor of actin polymerization [34], suggesting that the in vivo dosage needs to be considerably reduced to avoid excessive toxicity. In fact, cytochalasin B and the reduced congener DiHCB are both 20-fold less toxic than cytochalasin D in mice [35]. Nevertheless, cytochalasin D has demonstrated anticancer activity against multiple cell lines in vitro [29, 30], warranting further in vivo characterization. Although cytochalasins B and D have been examined in vivo against solid malignancies [4, 33], neither agent has been tested in vivo against disseminated hematological malignancies, and such data would be pivotal for characterizing the extent of cancers that might possibly be treated with microfilament-directed agents. Whether or not cytochalasin D would potentiate drug synergy with ADR, or other clinically approved chemotherapeutic agents is unclear, as the congener has yet to be examined for drug synergy. Therefore, it too is used in combination with ADR against P388/ADR leukemia in this study.

Knowledge of the effects of cytochalasins as single agents and optimization of any observed anticancer activity in vitro or in vivo is important in itself, and essential for the design and evaluation of studies involving the potential use of cytochalasin congeners as amplifiers of the activity of known chemotherapeutic agents in vivo. Therefore, this study seeks to determine: 1) whether cytochalasin B exerts potentially useful cytotoxicity on adherent carcinoma and melanoma, including sub-lines selected for metastatic capacity, as well as on suspension leukemia cells; 2) whether concomitant cytochalasin B/ADR, DiHCB/ADR, or cytochalasin D/ADR treatment against P388/ADR leukemia produces significant drug synergy; and 3) whether cytochalasins B and D are efficacious in vivo in prolonging the life of Balb/c mice challenged with P388/S and P388/ADR leukemias.

## Materials and methods

### Conversion of in vivo intradermal passaged M109 to continuous culture (M109c)

A cell suspension (1 × 10^6^ in 1 ml) derived from an in vivo passaged intradermal (i.d.) tumor was inoculated into 25 cm^2^ culture flasks, and suspended in 9 ml of RPMI 1640 complete medium containing 10 % newborn calf serum (GIBCO, Grand Island, NY), 0.4 units/ml penicillin, 0.4 μl/ml streptomycin, and 250 μg/ml fungizone. Flasks were incubated in 5 % CO_2_ at 37 °C for 6 days with one medium change at 3 days. For sub-culturing, the attached cells were trypsinized with 0.2 % trypsin-EDTA solution 10X (Sigma-Aldrich Corp., St. Louis, MO, USA) for 1 min at 37 °C, dislodged by a sharp knocking of the flasks during that period, washed, diluted to 10 ml with fresh complete medium, and 1 × 10^6^ cells seeded into 25 cm^2^ culture flasks (4 × 10^4^ cells/cm^2^). Floating colonies were observed during growth. These proved to be 20 % trypan blue negative, showed low tumorigenicity, and were not used.

After ten subculture passages over a period of 2 months, the attached M109 cells (M109c cells) achieved morphological stability and grew to confluency (2 × 10^5^ cells/cm^2^) in 6 days when subcultured at 4 × 10^4^ cells/cm^2^. Comparison between Balb/c mice challenged i.d. with M109c cells and matched groups challenged with in vivo passaged M109 cells showed that the challenges were indistinguishable in terms of growth kinetics, invasion, metastasis, and host survival. M109c retained in vitro and in vivo properties after 7 months of continuous passage in culture.

### Cytochalasin B preparation

Cytochalasin B was prepared from mold mattes of *Drechslera dematioidea* (ATCC 24346) as previously described [3, 4], and purified by preparative thin layer chromatography to greater than 99 % homogeneity after recrystallization from chloroform.

### 21, 22-dihydrocytochalasin B preparation

DiHCB was prepared by sodium-borohydride reduction of cytochalasin B in methanol at 25 °C. The product was recovered as a chloroform-soluble fraction and crystallized from benzene:hexane. DiHCB was compared to a commercially purchased sample of DiHCB (Sigma-Aldrich Corp.) and cytochalasin B (Poniard Pharmaceuticals, San Francisco, CA, USA) using reverse phase thin layer chromatography. The product was also characterized with ^1^H NMR spectroscopy (data not shown).

### Cytochalasin D preparation

Cytochalasin D was prepared from mold mattes of *Zygosporium masonii* (ATCC MYA­3308), and purified by preparative thin layer chromatography to greater than 99 % homogeneity after recrystallization from chloroform.

### Effect of cytochalasin B on cancer cell lines in vitro

The attached cell lines M109c, B16BL6, and B16F10 were seeded at 1 to 4 × 10^4^ cells/ml in 2 ml volumes in 24-well culture plates 1 day prior to treatment with cytochalasin B. Conditions for treatment of the attached cell lines were as detailed earlier for B16BL6 and B16F10 cells [4]. The suspension culture of P388/ADR cells was seeded at 5 × 10^4^ cells/ml and allowed to grow overnight before cytochalasin B treatment. Cells were treated with cytochalasin B for 3 h, as well as 2, 3, or 4 days. In the case of continuous exposure for 2, 3, or 4 days, attached cells were trypsinized and counted with a hemacytometer. Leukemia cell suspensions were counted with a Coulter Counter. In the case of short-term exposure, cells were washed twice with fresh medium, then trypsinized (except for P388/ADR cells), reseeded, and allowed to regrow for 3 days, at which time they were counted. Growth results were calculated as the number of cells generated above the seeding density compared to the untreated control cells and graphically presented as percent of control increase.

M109c clonogenic cells were determined by seeding aliquots containing 400–2000 trypsinized cells from each well into wells in another 24-well plate, culturing for 7 days, fixing in methanol (5 min), and staining with 0.1 % methylene blue (5 min). Colonies of greater than 10 cells were counted with a dissecting microscope.

### Determining the extent of drug synergy between cytochalasins and doxorubicin

To assess whether cytochalasin B, D or DiHCB synergizes with ADR, cells were treated with a cytochalasin congener for 2.5 h over a concentration range of 0 to 150 μM, followed by ADR over a concentration range of 0 to 9 μM for 3 h. IC_50_ and IC_80_ values were taken at a series of concentrations for each chemotherapeutic agent in order to construct an isobologram. IC_50_ and IC_80_ values for the single agents and for combinations were determined by MTT (3-(4,5-dimethylthiazol-2-yl)-2,5-diphenyltetrazolium bromide) assays.

In addition, drug synergy was assessed by clonogenic assays in which P388/ADR cells were seeded in 24-well plates at early log phase. Cytochalasin B or DiHCB were administered for 2.5 h, followed by ADR for 3 h at a series of concentrations. Aliquots of the treated cells were then removed and cloned in soft agar in additional 24-well plates. Results from the assays were plotted as log surviving fractions at a given cytochalasin concentration as a function of ADR concentration. Fold-synergism was then calculated at relatively low concentrations of cyotchalasin where cytochalasin B or DiHCB-alone had minimal influence on cloning efficiency.

### P388 leukemias in vivo

For chemotherapy testing, Balb/c mice under isoflurane anesthesia (Sigma-Aldrich Corp.) were challenged with 2 × 10^5^ trypan blue negative P388/S or P388/ADR cells subcutaneously (s.c.) in a volume of 200 μl. Untreated mice were kept in order to determine the lethality of the challenge without chemotherapeutic intervention. Long-term survival was defined as challenged mice that survived the duration of the observation period.

### Cytochalasins B and D intraperitoneal administration

Cytochalasins B and D were prepared in suspension form in 2 % carboxymethyl cellulose 1 % tween 20 (CMC/Tw) for intraperitoneal (i.p.) administration, as previously described [3, 4]. The congeners or the vehicle were administered to leukemia-challenged mice on Days 1–8 following the initial challenge.

### Statistics

Survival analysis used the Cox-Mantel test as detailed by Lee [36]. Test of hypotheses for between subjects effects were applied, and of time vs. group interactions using the Geisser-Greenhouse adjustment in cases where tests of orthogonal components showed absence of sphericity.

## Results

### Effects of cytochalasin B treatment in vitro on M109c, P388/ADR, B16BL6, and B16F10

**Fig. 1:**
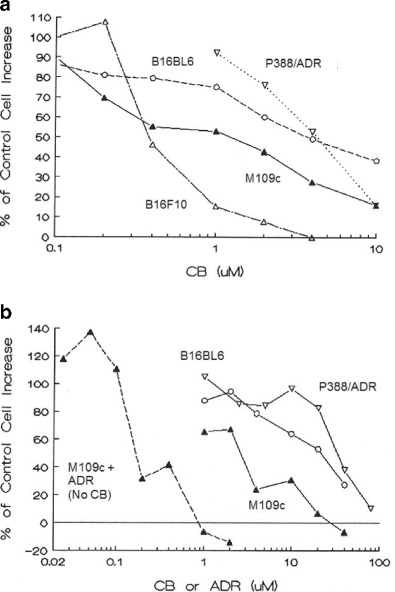
Cytochalasin B against multiple murine cancer cell lines in vitro. **a** Effects of 3-day continuous exposure to cytochalasin B on growth of attached and suspension culture murine neoplastic cell lines. Continuous cell lines as indicated in the figure were seeded at 4 to 8 × 10^4^ cells per well (2 to 4 × 10^4^ cells/cm^2^) for attached cells in 2 ml (triplicate, 24 well-plates, complete medium; see materials and methods), and were allowed to attach overnight. P388/ADR cells were seeded at 5 × 10^4^ cells/ml in 2 ml of medium, and allowed to grow overnight. Cells were continuously exposed to cytochalasin B (CB) at concentrations shown for 3 days at 37 °C in 5 % CO_2_ in a humidified atmosphere. Attached cells (B16BL6, B16F10, and M109c) were washed, trypsinized, and counted with a hemacytometer. P388/ADR suspensions were counted with a Coulter counter. The increase in cell number beyond the number present at the time of treatment initiation was compared to the increase in cell number in the corresponding controls. **b** Effects of short-term, 3 h exposure to cytochalasin B on growth of attached and suspension culture neoplastic cell lines. Cell lines were seeded as detailed in Fig. 1a, but treated with CB at the concentrations shown for only 3 h

The effects of continuous exposure to cytochalasin B for 3 and 4 days in vitro were determined for M109c and compared to values obtained with two other attached murine tumor lines, B16F10 and B16BL6 melanoma, and with a suspension culture of murine P388/ADR leukemia cells. The effects of 3 day exposure are shown in Fig. 1a, while the IC_50_ and IC_80_ values for 3 h, as well as for 2, 3, and 4 day exposures are presented in Table 1. B16BL6 and P388/ADR are relatively resistant to CB treatment (IC_50_ values approximately 5 uM for 3-day exposure). M109c shows an IC_50_ after 3 days of exposure of 2 μM. The remarkable sensitivity of the B16F10 line which is selected by repeated in vivo subculture for ten sequential challenges from metastatic foci of B16BL6 malignant melanoma is striking and potentially important. The IC_50_ for B16F10 melanoma is 0.4 μM, fully ten-fold more cytotoxic than for the non-selected original B16BL6 line. This may point to special efficacy of microfilament-directed agents for highly metastatic selected cell lines.

**Table 1:** Effects of cytochalasin B against murine cancer lines in vitro

CB exposure time	3 h		2 days		3 days		4 days	
IC concentrations (μM)	IC_50_	IC_80_	IC_50_	IC_80_	IC_50_	IC_80_	IC_50_	IC_80_
M109c	2.56	12.23	0.83	6.02	0.42	3.77	0.25	0.75
B16F10	ND	ND	0.80	3.50	0.46	1.59	0.37	1.21
B16BL6	10.46	44.86	2.10	20.04	0.96	17.03	0.87	10.41
P388/ADR	105.5	188.4	6.23	10.45	4.24	6.47	ND	ND
P388/S	51.9	84.1^a^	3.10	5.02	ND	ND	ND	ND

*ND* Not determined, ^a^2.5 h

### Effects of cytochalasin B treatment for 3 h in vitro on P388/ADR, M109c, and B16BL6

Since in vivo tolerated exposure to bioactive concentrations of cytochalasin B administered systemically in the absence of sustained release formulation is likely to be a matter of hours rather than days [3, 4], we examined the short-term (3 h) effects of cytochalasin B on P388/ADR, M109c and B16BL6 cells (Fig. 1b). The IC_50_ and IC_80_ values are presented in Table 1, and are compared with values obtained for the P388/ADR line, as well as the parental drug-sensitive P388 line we have previously determined (unpublished data). P388/ADR and B16BL6 show potent resistance to cytochalasin B with 3-hour IC_50_ values in the range of 30 μM. M109c showed a 10-fold higher sensitivity to cytochalasin B with an IC_50_ after 3 h of 3 μM. The comparative toxicities of cytchalasin B and ADR against M109c cells in a 3 h exposure are also presented in Fig. 1b. It is apparent that cytochalasin B is 14-fold less cytotoxic in terms of comparative IC_50_ values against M109c cells than is ADR, confirming the comparatively low cytotoxicity of short-term cytochalasin B exposure.

### Extent of drug synergy between cytochalasins and doxorubicin against P388/ADR leukemia

**Fig. 2:**
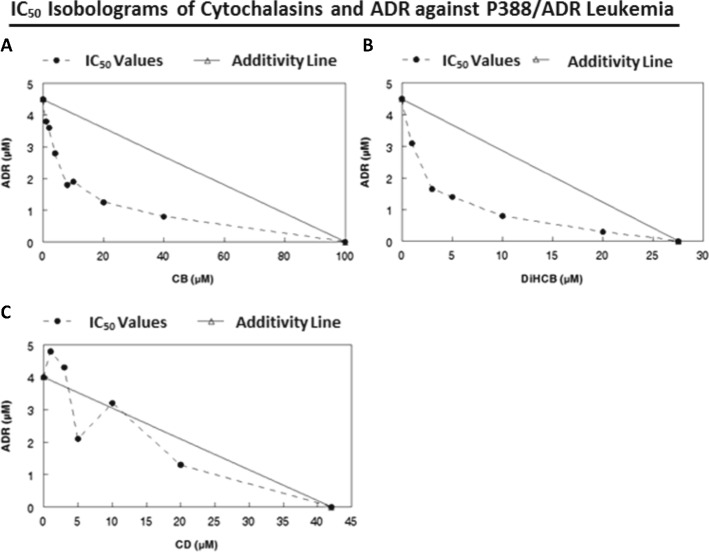
IC_50_ Isobolograms of cytochalasins and doxorubicin to determine extent of drug synergy against P388/ADR leukemia. **a** CB and ADR. **b** DiHCB and ADR. **c** CD and ADR

**Fig. 3:**
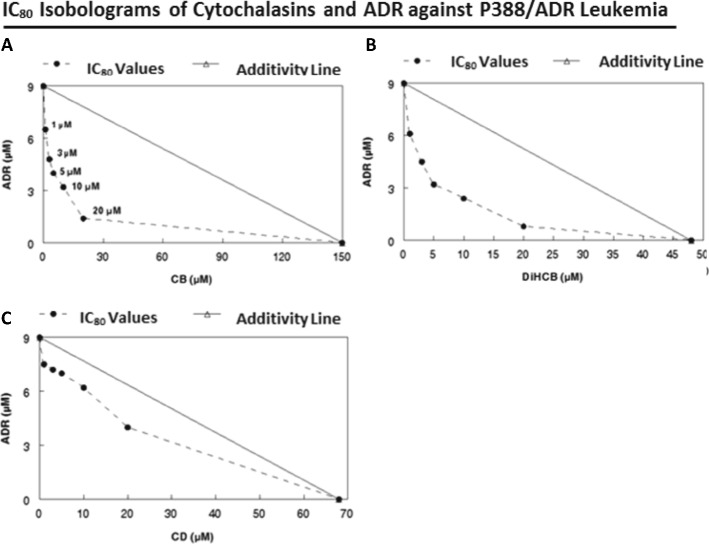
IC_80_ Isobolograms of cytochalasins and doxorubicin to determine extent of drug synergy against P388/ADR leukemia. **a** CB and ADR. **b** DiHCB and ADR. **c** CD and ADR

**Fig. 4:**
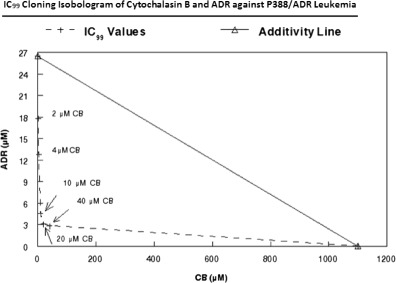
IC_99_ Cloning isobologram of cytochalasin B and doxorubicin to determine extent of drug synergy against P388/ADR leukemia. Values of cytochalasin B are provided in the figure to clarify each point

Despite the lower activity of cytochalasin B in P388/ADR leukemia, it still produced a substantial synergistic effect with ADR against the ADR-resistant cell line, as assessed by the IC_50_ (Fig. 2a) and IC_80_ (Fig. 3a) isobolograms. While low concentrations of cytochalasin B demonstrated marked synergy in the IC_80_ isobologram, it took a 150 μM concentration for cytochalasin B by itself to reach the IC_80_ inhibition point. (Fig. 3a). This pattern was also seen in the IC_99_ cloning isobologram for cytochalasin B/ADR (Fig. 4) where 1.1 mM cytochalasin B is required to reach the IC_99_ value with the single agent treatment. DiHCB also demonstrated considerable drug synergy with ADR against P388/ADR leukemia, with a smooth curve well under the additivity line being observed in both the IC_50_ and IC_80_ isobolograms (Figs. 2b and 3b). Cytochalasin D demonstrated little to no synergy with ADR against P388/ADR leukemia (Figs. 2c and 3c). Nevertheless, cytochalasin D appeared to be intermediate between DiHCB and cytochalasin B in regards to inhibiting growth as single agents at their IC_50_ and IC_80_ values. DiHCB produced IC_50_ and IC_80_ inhibitions at 28 μM and 48 μM respectively. Cytochalasin B required 100 μM and 150 μM to reach IC_50_ and IC_80_. Cytochalasin D required 42 μM and 68 μM to reach IC_50_ and IC_80_ respectively. (Figs. 2 and 3).

**Fig. 5:**
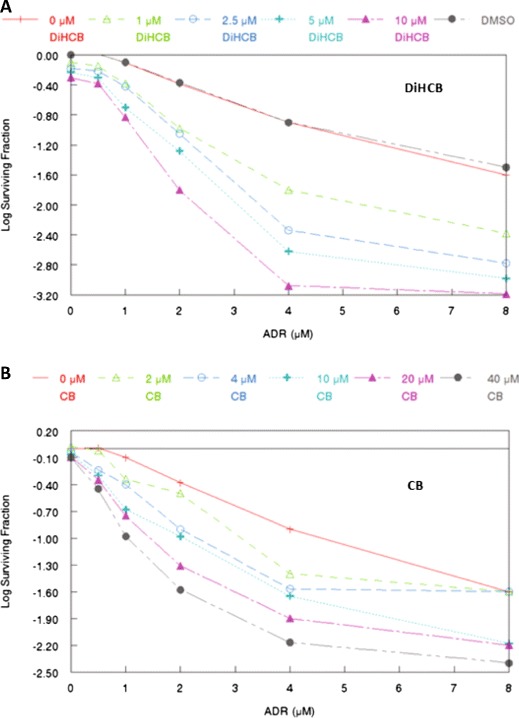
Comparison of doxorubicin drug synergy between cytochalasin B and 21, 22-dihydrocytochalasin B against P388/ADR leukemia assessed by log surviving fraction cloning assays. **a** Cloning assay for concomitant DiHCB/ADR treatment. **b** Cloning assay for concomitant CB/ADR treatment. Concomitant treatments were administered at varying concentrations for 3 h

The extent of drug synergy potentiated by cytochalasin B and DiHCB was further evaluated in log surviving fraction cloning assays comparing the parent compound (cytochalasin B) against its reduced congener (DiHCB) in eliciting drug synergy with ADR (Fig. 5). Differences in the extent of drug synergy were readily apparent. DiHCB appeared to synergize much more effectively with ADR, as it produced a lower log surviving fraction at much lower concentrations (1 to 10 μM) than did cytochalasin B (2 to 40 μM). For example, at 8 μM ADR, 40 μM cytochalasin B produced a log fraction of −2.4 against P388/ADR leukemia cells. By contrast, only 10 μM DiHCB was needed to produce a log fraction of −3.19 when combined with the same concentration of ADR. Therefore, a lower concentration of DiHCB was able to elicit a stronger synergistic effect than did its oxidized congener.

### Effects of cytochalasins B and D against P388 leukemias in vivo

**Fig. 6:**
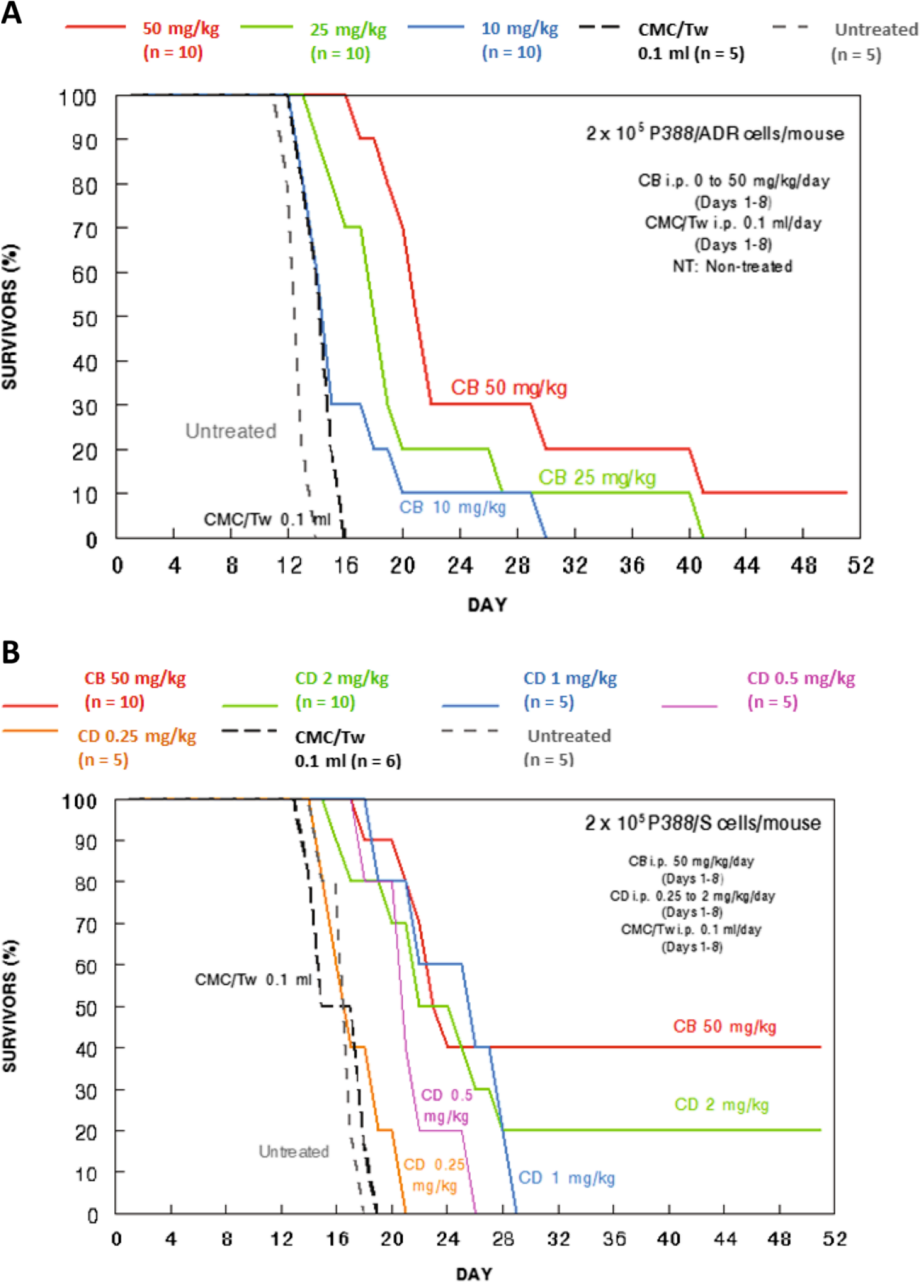
Cytochalasins against P388/ADR and P388/S murine leukemias in vivo. **a** Effects of intraperitoneally administered cytochalasin B on the survival of P388/ADR murine leukemia-challenged mice. Cytochalasin B was administered at 10, 25, or 50 mg/kg, while the CMC/Tw vehicle was administered at 0.1 ml. The treatments were administered on Days 1–8 following the leukemia challenge. **b** Effects of intraperitoneally administered cytochalasin B or D on the survival of P388/S murine leukemia-challenged mice. Cytochalasin B was administered at 50 mg/kg, and cytochalasin D was administered at 0.25, 0.5, 1, or 2 mg/kg. The CMC/Tw vehicle was again administered at 0.1 ml. The treatments were administered on Days 1–8 following the leukemia challenge. The number of mice per each treatment group is provided in both panels. Survival of mice in all treatment groups was significantly different from controls left untreated or treated only with the vehicle (CMC/Tw) in both panels; *p* < 0.05, as assessed by a Cox-Mantel test

Cytochalasin B appeared to increase the life expectancy of Balb/c mice challenged with either P388/ADR or P388/S leukemias (Fig. 6). It was discerned from the P388/ADR protocol that Balb/c mice could take up to 50 mg/kg/day i.p. for eight consecutive days (Days 1–8). Therefore, only this dose was examined for P388/S challenged mice, as the antitumor activity of cytochalasin B appeared to be dose dependent. Interestingly, 50 mg/kg cytochalasin B was able to produce 10 % long-term survival in the multidrug resistant P388/ADR cohort, and 40 % long-term survival in the drug sensitive P388/S cohort.

The antitumor effects of cytochalasin B were mirrored by cytochalasin D at much lower concentrations (Fig. 6b). It only took 2 mg/kg/day cytochalasin D administered for eight consecutive days (Days 1–8) to produce marked prolongation in the life expectancy of mice challenged with P388/S, as well as a 20 % long-term survival rate. Whether or not cytochalasin D would exhibit the same antitumor effect against P388/ADR at these lower concentrations remains unclear, as not enough mice remained to establish another treatment group of sufficient quantity. Nevertheless, it is likely that at least some prolongation in life expectancy would be observed. The cytochalasin vehicle CMC/Tw did not affect the life span of mice challenged with either leukemia, demonstrating that there is no effect of the lipophilic detergent vehicle on the leukemia challenges in the absence of cytochalasins.

## Discussion

In the present study, we have demonstrated that cytochalasin B is 14-fold less cytotoxic against M109c cells than is ADR in a 3 h exposure assay when the washed cells are allowed to regrow, and the respective IC_50_ values are evaluated. These data further support that cytochalasin B is only moderately cytotoxic [3, 4]. It is worth noting that cytochalasin B is relatively more cytotoxic in vitro against attached cell lines (Table 1 and Fig. 1) than against a suspension culture, particularly in 3 h drug comparisons. It should also be noted that among the attached neoplastic cell lines, there is variable sensitivity to cytochalasin B, indicating that the effects of the agent in vitro are not solely related to nonspecific alterations in adherence to the substrate. In particular, cytochalasin B was much more cytotoxic against B16F10 than B16BL6. B16F10 is a highly metastatic variant of B16 murine melanoma that is selected for its propensity to metastasize to the lungs by serial passaging beginning with B16BL6, and then passaging from lung metastases ten times in vivo [37–39]. We have previously demonstrated that cytochalasin B is markedly cytotoxic against B16F10 in vitro [4], and this direct comparison against a less metastatic variant (B16BL6) intriguingly suggests that cytochalasin B may have a profound inhibitory effect on highly metastatic cells.

Cytochalasin B and DiHCB-mediated drug synergy warrant further investigation based on the in vitro data. As assessed by isobolographic analysis, it was apparent that cytochalasin B and DiHCB synergize with ADR against ADR-resistant P388/ADR leukemia (Figs. 2 and 3), a cell line in which neither of the cytochalasin congeners, nor ADR show substantial inhibitory activity alone. DiHCB/ADR treatments (Fig. 5a) were able to produce a lower log surviving fraction of P388/ADR leukemia cells than was the combination of cytochalasin B/ADR (Fig. 5b), showing that DiHCB synergizes more efficiently with ADR than does cytochalasin B. These data also indicate that cytochalasin synergy with ADR is not dependent on inhibiting glucose transport, as DiHCB, which does not affect glucose transport was a more effective synergizing agent. Nevertheless, both compounds substantially improved the efficacy of ADR treatments in an ADR-resistant cell line, warranting further investigation of concomitant cytochalasin therapies. In fact, cytochalasin B has already been shown to synergize with cytarabine [40] and vincristine [41], suggesting that this, and potentially other cytochalasin congeners, may be used to supplement current chemotherapeutic protocols.

This study marks the first time that either cytochalasin B or D was shown to have significant antitumor activity against leukemia in vivo. Although P388/ADR leukemia is known to be a multidrug resistant cell line [42–44], cytochalasin B was still able to exert marked antitumor activity (Fig. 6a), demonstrating that the novel mechanisms by which the compound perturbs neoplastic cells can inhibit the progression of disseminated hematological cancers. As expected, more antitumor activity was observed in mice challenged with drug sensitive P388/S leukemia, and nearly half of the treatment group achieved long-term survival when treated with 50 mg/kg/day i.p. cytochalasin B for eight consecutive days (Fig. 6b). Due to the increased cytotoxicity of cytochalasin D, only 2 mg/kg/day i.p. for the same time period could be used in mice, but this lower dose level was still able to exert substantial antitumor effects, with 20 % of the treatment group achieving long-term survival (Fig. 6b).

From these data, it can be surmised that cytochalasins exert substantial anticancer activity in vitro and in vivo, and could likely be used in combination with currently approved antineoplastic agents. We have already demonstrated that cytochalasin B elicits marked antitumor activity against murine M109 lung carcinoma and murine B16 melanoma in vivo [3]. The in vivo effects of cytochalasin B and a liposome encapsulated derivative are further characterized against M109 lung carcinoma in the subsequent paper of this series [45], providing additional evidence for the clinical potential of microfilament-directed agents.
